# Resilience and Social Support Improve Mental Health and Quality of Life in Patients with Post-COVID-19 Syndrome

**DOI:** 10.3390/ejihpe14010015

**Published:** 2024-01-15

**Authors:** Ioannis Moisoglou, Aglaia Katsiroumpa, Antigoni Kolisiati, Irene Vraka, Katerina Kosiara, Olga Siskou, Daphne Kaitelidou, Olympia Konstantakopoulou, Theodoros Katsoulas, Parisis Gallos, Petros Galanis

**Affiliations:** 1 Faculty of Nursing, University of Thessaly, 41500 Larissa, Greece; 2 Clinical Epidemiology Laboratory, Faculty of Nursing, National and Kapodistrian University of Athens, 11527 Athens, Greece; aglaiakat@nurs.uoa.gr (A.K.); katerina_kosiara@yahoo.gr (K.K.); pegalan@nurs.uoa.gr (P.G.); 3 Department of Endocrinology and Diabetes Center, General Hospital “G. Gennimatas”, 11527 Athens, Greece; antigonikol@outlook.com; 4 Department of Radiology, P. & A. Kyriakou Children’s Hospital, 11527 Athens, Greece; irenevraka@yahoo.gr; 5 Department of Tourism Studies, University of Piraeus, 18534 Piraeus, Greece; olsiskou@nurs.uoa.gr; 6 Center for Health Services Management and Evaluation, Faculty of Nursing, National and Kapodistrian University of Athens, 11527 Athens, Greece; dkaitelid@nurs.uoa.gr (D.K.); olykonstant@nurs.uoa.gr (O.K.); 7 Faculty of Nursing, National and Kapodistrian University of Athens, 11527 Athens, Greece; tkatsoul@nurs.uoa.gr (T.K.); parisgallos@nurs.uoa.gr (P.G.)

**Keywords:** post-COVID-19 syndrome, resilience, social support, quality of life, mental health

## Abstract

Physical and mental health problems among post-COVID-19 patients are common, even a year after infection. As there is no prior study available, we investigated the impacts of resilience and social support on anxiety, depression, and quality of life among patients with post-COVID-19 syndrome. We conducted a cross-sectional study with a convenience sample. The measures included the demographic and clinical characteristics of patients, the Brief Resilience Scale, the Multidimensional Scale of Perceived Social Support, the Patient Health Questionnaire-4 (PHQ-4), and the EuroQol-5D-3L. The mean age of patients was 44.8 years. The total PHQ-4 score suggested that 32.8% of patients with post-COVID-19 syndrome experienced severe psychological distress, 32.8% experienced moderate distress, 23% experienced mild distress, and 11.5% had no distress. Moreover, 60.7% of patients had anxiety scores of ≥3 and 69.7% had depression scores of ≥3, indicating possible major anxiety or depression disorder. The mean EQ-5D-3L index value was 0.36, and the mean EQ-5D-3L VAS was 54.1. Multivariable analysis identified that resilience and social support reduced anxiety and depression among patients. Also, we found a significant positive relationship between resilience and social support, and quality of life. Our findings suggest that resilience and social support can be protective by reducing anxiety and depression and improving quality of life among patients with post-COVID-19 syndrome. Policymakers should develop and implement healthcare management programs to provide psychological support to these patients.

## 1. Introduction

Post-COVID-19 syndrome, also known as long COVID-19, is defined by the World Health Organization (WHO) as a condition where symptoms are present three months after SARS-CoV-2 infection and cannot be explained by alternative diagnoses [1]. Fatigue, shortness of breath, sleep disorders, and cognitive dysfunction are the most common symptoms in patients with post-COVID-19 syndrome, while over 200 long-term different symptoms are identified in the literature [2,3]. A large proportion of COVID-19 patients also have post-COVID-19 syndrome. For example, according to a prediction model by the WHO, more than 17 million people across the WHO European Region may have experienced post-COVID-19 syndrome during the first two years of the pandemic [4]. Moreover, a meta-analysis with a total of 1.2 million symptomatic COVID-19 cases found that the fatigue cluster of long COVID-19 occurred in 51.0% of cases, the respiratory cluster occurred in 60.4% of cases, and the cognitive cluster occurred in 35.4% of cases [5]. Additionally, at >12-month follow-up, 41%, 31%, 30%, 22%, and 22% of confirmed SARS-CoV-2 cases continued to experience fatigue, dyspnea, sleep disorder, myalgia, and cognitive impairment, respectively [3,6]. 

The impact of post-COVID-19 syndrome on the mental health of patients is high. The European Centre for Disease Prevention and Control (ECDC) performed a meta-analysis and found that the prevalence of anxiety, depression, and post-traumatic stress disorder in patients with post-COVID-19 syndrome recruited in community settings was 17.2%, 17.3%, and 20.6%, respectively [7]. The prevalence of mental health issues was even higher for patients recruited in hospital settings, i.e., the prevalence of anxiety was 27.5%, and that of depression was 23.3%. A similar systematic review found that anxiety (ranging from 6.5% to 63% across the studies), depression (ranging from 4% to 31%), and post-traumatic stress disorder (ranging from 12.1% to 46.9%) were the most common mental health issues in patients with post-COVID-19 syndrome [8]. Even young people at 3 months post-COVID-19 showed emotional vulnerability in higher psychological distress (depression, anxiety, and stress) and dysphoria signs (irritability, discontent, interpersonal resentment, and feelings of renunciation/surrender) [9].

Moreover, post-COVID-19 syndrome affects patients’ quality of life since a meta-analysis found that the prevalence of poor quality of life was 59% [10]. Additionally, among patients with post-COVID-19 syndrome, 41.5% reported extreme pain/discomfort, 37.5% reported extreme anxiety/depression, 36% reported extreme problems with mobility, 28% reported extreme problems with usual activities, and 8% reported extreme problems with self-care [10]. 

The positive impact of resilience and social support during the pandemic has already been proven. In particular, a meta-analysis found a significant negative relationship between resilience and psychological distress among COVID-19 patients [11]. Moreover, a review including workers found that resilience was associated with lower levels of anxiety, depression, and burnout [12]. Similarly, a systematic review found that resilience and social support among healthcare workers improved mental and psychological health outcomes [13]. Additionally, low perceived social support was a risk factor for adverse psychological states in COVID-19 patients, such as stress, anxiety, and depression [14]. Also, social support seemed very important during the first wave of the pandemic due to its negative correlation with psychological pressure, mental discomfort, and anxiety [15].

Until now, research has only explored the relationship between a few demographic and clinical characteristics of patients with post-COVID-19 syndrome and mental health and quality of life. Greater anxiety and depression were reported in female patients, patients with a psychiatric history, and patients with intensive care unit admission [8,16]. Moreover, quality of life was lower among patients admitted to intensive care and patients with higher levels of fatigue [10]. However, the available studies have not investigated the influence of psychosocial factors on patients’ lives. Thus, the aim of our study was to explore the impacts of resilience and social support on anxiety, depression, and quality of life among patients with post-COVID-19 syndrome.

## 2. Materials and Methods

### 2.1. Study Design and Population

We conducted an online cross-sectional study in Greece between November 2022 and January 2023. First, we created an online version of the study questionnaire using Google Forms. Then, we posted the questionnaire on the Facebook page of the long COVID Greece patient society after administrators’ approval [17]. Patients could participate in our study by clicking on the link to the questionnaire. Therefore, we recruited patients with post-COVID-19 syndrome via convenience sampling. The response rate was unknown since we could not measure the number of patients who clicked on the link of the study questionnaire but did not complete it. The Long COVID Greece patients society is a member of a European network of long COVID-19 patient associations, i.e., Long COVID Europe [18]. The Long COVID Greece patients society is a non-profit organization and was created by patients with post-COVID-19 syndrome. 

Participation in our study was allowed if the following criteria were met: adults aged ≥18 years; individuals who understand the Greek language since the study questionnaire was in Greek; previous confirmed SARS-CoV-2 infection with a molecular test; patients who presented symptoms and signs consistent with COVID-19 for ≥12 weeks after the COVID-19 diagnosis and were not attributable to alternative diagnoses in order to meet the definition of post-COVID-19 syndrome [1,19]. 

The minimum sample size required 121 patients considering a low effect size (f^2^ = 0.11), the power as 95%, the alpha level as 5%, and the number of independent variables as 10 [20].

### 2.2. Demographic and Clinical Characteristics

We measured gender (females or males), age (continuous variable), hospitalization in a COVID-19 ward (no or yes), hospitalization in a COVID-19 intensive care unit (no or yes), the duration of COVID-19 symptoms (continuous variable), anxiety disorders before post-COVID-19 syndrome (no or yes), and depression before post-COVID-19 syndrome (no or yes).

### 2.3. Measures

#### 2.3.1. Resilience

We measured patients’ resilience with the Brief Resilience Scale (BRS) [21]. The BRS consists of six items, e.g., “I tend to bounce back quickly after hard times”. Answers are on a 5-point Likert scale, i.e., 1: strongly disagree, 2: disagree, 3: neutral, 4: agree, and 5: strongly agree. The total score is calculated by dividing the total sum by the total number of questions answered. Thus, a total score from 1 to 5 is produced, with higher values indicating higher levels of resilience. 

The BRS was translated and validated in the Greek language [22]. In our study, Cronbach’s coefficient alpha for the BRS was 0.878.

#### 2.3.2. Social Support

We used the Multidimensional Scale of Perceived Social Support (MSPSS) to measure family support, friend support, and significant other support [23]. The MSPSS consists of 12 items, and answers are on a seven-point Likert scale from strongly disagree to strongly agree. Each factor includes four items and takes a total score from 1 to 7. Improved scores in the MSPSS indicate a higher level of support. 

We used a reliable and valid Greek version of the MSPSS in our study [24]. We found that Cronbach’s coefficient alpha for the factor “family support” was 0.968, the factor “friends support” was 0.971, and the factor “significant others support” was 0.919.

#### 2.3.3. Mental Health Outcomes

We used the Patient Health Questionnaire-4 (PHQ-4) to measure anxiety and depression among patients with post-COVID-19 syndrome [25]. The PHQ-4 consists of four items: two items measure anxiety, and two items measure depression. The answers to items take values from 0 (not at all) to 3 (almost every day). Thus, the total score on the anxiety and depression scales ranges from 0 to 6. Also, the total score on the PHQ-4 is calculated with values from 0 to 12. Higher values indicate higher levels of anxiety and depression. Individuals with a total score from 0 to 2 are rated as normal, those with a total score from 3 to 5 are considered as experiencing mild distress, those with a total score from 6 to 8 are considered as experiencing moderate distress, and those with a total score from 9 to 12 are considered as experiencing severe distress. Moreover, a total anxiety or depression score of ≥3 is indicative of possible major anxiety or depression disorder. 

We used the reliable and valid Greek version of the PHQ-4 [26]. In our study, Cronbach’s coefficient alpha for the PHQ-4 was 0.864, that for the anxiety scale was 0.854, and that for the depression scale was 0.849.

#### 2.3.4. Quality of Life

We used the 3-level version of EQ-5D (EQ-5D-3L) to measure patients’ quality of life [27]. The EQ-5D-3L includes the EQ-5D descriptive system and the EQ visual analog scale (EQ VAS). The EQ-5D descriptive system includes five dimensions (i.e., mobility, self-care, usual activities, pain/discomfort, and anxiety/depression), and each of them has three levels (i.e., no problems, some problems, and extreme problems). The answers regarding the five dimensions result in a 5-digit number that describes the individual’s health state. This 5-digit number is then converted into a single summary index value using a set of weights which are produced from representative samples of people from the general population. We used the set of weights that were produced from a study in Greece [28]. Higher EQ-5D-3L index values indicate higher levels of quality of life. Moreover, individuals can self-rate their health status on the EQ-VAS, which is a vertical visual analog scale with values from 0 (the worst imaginable health state) to 100 (the best imaginable health state). In our study, Cronbach’s coefficient alpha for the EQ-5D-3L was 0.791.

### 2.4. Ethical Issues

Patients with post-COVID-19 syndrome could participate in this study on an anonymous and voluntary basis. Moreover, we did not collect personal information data, e.g., name, insurance number, IP address, email address, etc. We informed patients about the study’s aim and design on the first page of the online questionnaire. Then, patients who gave their informed consent could access the full version of the questionnaire.

Our study protocol was approved by the Ethics Committee of the Faculty of Nursing, the National and Kapodistrian University of Athens (reference number; 420, 10 October 2022). Additionally, we applied the guidelines of the Declaration of Helsinki. 

### 2.5. Statistical Analysis

We used numbers and percentages to present categorical variables. Moreover, we used means, standard deviation, medians, minimum values, and maximum values to present continuous variables. Resilience, family support, friend support, and significant other support were the independent variables, while anxiety, depression, and quality of life were the dependent variables. We considered the demographic and clinical characteristics of patients with post-COVID-19 syndrome as possible confounders. We used the Kolmogorov–Smirnov test and Q–Q plots to check the normality of the continuous variables. Because the dependent variables followed a normal distribution, we applied linear regression analysis in order to find out the impacts of predictors. First, we performed a univariate linear regression analysis and then we constructed a multivariable linear regression model in order to eliminate confounding factors. In that case, we present unadjusted and adjusted beta coefficients, 95% confidence intervals (CIs), and *p*-values. We did not include hospitalization in a COVID-19 intensive care unit in the linear regression analysis due to the limited variability, i.e., only four patients had been hospitalized in a COVID-19 intensive care unit. Independent variables with a *p*-value of <0.05 in the final multivariable model were considered statistically significant. We used IBM SPSS 21.0 (IBM Corp. Released 2012, IBM SPSS Statistics for Windows, Version 21.0. Armonk, NY, USA: IBM Corp.) for the analysis.

## 3. Results

The study population included 122 patients with post-COVID-19 syndrome with a mean age of 44.8 years. Most of them were females (73%). Among patients, 17.2% had been hospitalized in a COVID-19 ward and 3.3% in a COVID-19 intensive care unit. The mean duration of COVID-19 symptoms was 11.6 months. Among patients, 18.9% reported that they had been diagnosed with an anxiety disorder and 11.5% with depression before post-COVID-19 syndrome. The demographic and clinical characteristics of patients with post-COVID-19 syndrome are presented in Table 1.

Descriptive statistics for the scales in our study are shown in Table 2. The total PHQ-4 score suggested that 32.8% (n = 40) of patients with post-COVID-19 syndrome experienced severe psychological distress, 32.8% (n = 40) experienced moderate distress, 23% (n = 28) experienced mild distress, and 11.5% (n = 14) had no distress. Moreover, 60.7% (n = 74) of patients had anxiety scores of ≥3, and 69.7% (n = 85) had depression scores of ≥3, indicating possible major anxiety or depression disorder.

The mean EQ-5D-3L index value was 0.36, and the mean EQ-5D-3L VAS was 54.1. The mean resilience score was 3.32, indicating a moderate level of resilience. Patients received more support from significant others (mean = 5.73) than family (mean = 5.41) and friends (mean = 5.22).

Multivariable linear regression analysis identified that increased resilience (adjusted beta = −0.56; 95% CI = −0.94 to −0.17) and significant others’ support (adjusted beta = −0.34; 95% CI = −0.64 to −0.03) reduced anxiety (Table 3). Also, we found that resilience (adjusted beta = −0.42; 95% CI = −0.80 to −0.05) and significant others’ support (adjusted beta = −0.39; 95% CI = −0.69 to −0.09) reduced depression in patients with post-COVID-19 syndrome (Table 4).

Multivariable linear regression analysis with quality of life as the dependent variable is shown in Table 5 and Table 6. We found that increased resilience (adjusted beta = 0.10; 95% CI = 0.02 to 0.18) and significant other support (adjusted beta = 0.10; 95% CI = 0.04 to −0.16) were associated with increased EQ-5D-3L index values. Moreover, significant other support (adjusted beta = 5.55; 95% CI = 1.59 to 9.51) improved the EQ-5D-3L VAS.

## 4. Discussion

The impact of the COVID-19 pandemic on the mental health of citizens has been significant. Fear of infection, quarantine, disruption of the educational process, and teaching via e-learning were some of the factors that affected mental health, making it imperative to modify mental health services to meet the needs of patients. Patients who experienced long COVID-19 may have already had compromised mental health [29,30,31]. However, the literature on factors that influence the mental health and quality of life in patients with post-COVID-19 syndrome is poor. The majority of studies focused only on the measurement of mental health and quality of life among patients. So far, a small number of demographic and clinical characteristics of patients have been studied. To the best of our knowledge, our study is the first that investigates the role of psychosocial factors on mental health and quality of life among patients with post-COVID-19 syndrome. In particular, we explored the relationship between resilience and social support, and mental health and quality of life in patients with post-COVID-19 syndrome.

Quality of life among patients with post-COVID-19 syndrome in our study was very poor. In our study, the EQ-5D-3L index value was 0.36, while the EQ-5D-3L index norm values based on the Greek value test were 0.916 in the 45–54 group, 0.739 in the ≥75 group, and 0.913 in all age groups [32]. Moreover, the EQ-5D-3L VAS in our patients was 54.1, while the EQ-5D-3L VAS norm values were 78 in the 45–54 group, 54 in the ≥75 group, and 79 in all age groups [28]. We compared our results with the 45–54 group since the mean age of our sample was 44.8 years. Thus, the quality of life of our patients was even worse than that of the Greek elderly over 75 years old. Additionally, similar studies in Spain, France, the USA, and China found that the EQ-5D-3L VAS among patients with post-COVID-19 syndrome ranged from 64 to 80, while the EQ-5D-3L index value ranged from 0.71 to 0.86 [33,34,35].

Moreover, our patients experienced high levels of anxiety and depression. In particular, the proportion of our patients reporting possible major anxiety (60.7%) and depression disorder (69.7%) was much higher than that reported in a meta-analysis (the overall prevalence of anxiety was 23% and that of depression was 17%) [36]. Additionally, another meta-analysis including only studies with low/moderate risk of bias found that the overall prevalence of depression among patients recruited in the community setting was 17.3%, while the prevalence of the disease among patients recruited in the hospital setting was 23.3% [7]. Moreover, a systematic review found that the frequency of depressive symptoms ranged from 11 to 28%, while the frequency of clinically significant depression ranged from 3 to 12% [16].

Our results show a significant negative relationship between resilience and anxiety and depression. In other words, the higher a patient’s resilience, the lower their anxiety and depression. Moreover, our multivariable model identified that resilience increased quality of life in our sample. The literature confirms the positive effect of resilience on individuals’ lives. In particular, a meta-analysis found a significant negative correlation between resilience and psychological distress in COVID-19 patients [11]. Similarly, a meta-analysis including patients with a somatic illness or health problem found a negative relationship between resilience and anxiety and depression [37]. Additionally, a meta-analysis including samples from the general population found a positive correlation between trait resilience and positive indicators of mental health, and a negative correlation between trait resilience and negative indicators of mental health [38]. Moreover, it is well known that during the COVID-19 pandemic, resilience helped people, especially vulnerable groups (e.g., the elderly or patients), to maintain a good quality of life [39,40,41,42,43].

Resilience is defined as an individual’s ability to withstand or recover quickly from difficult conditions [38]. In other words, resilience can be described as a defense mechanism, which gives people the ability to cope successfully with stressful experiences and bounce back from negative experiences [44]. Additionally, resilience implies the flexible use of emotional resources, such as flexibility, perseverance, balance, and self-reliance for adapting to adversity [37,45]. Therefore, resilience is crucial in promoting individuals’ positive mental health, especially during the COVID-19 pandemic where mental health was compromised in several ways (e.g., lockdowns, quarantine measures, social isolation, loneliness, etc.) [46,47,48,49]. Moreover, several studies showed that higher levels of resilience were associated with better mental health, not only in the general population but also in COVID-19 patients [50,51,52,53,54]. Thus, higher levels of resilience among patients with post-COVID-19 syndrome can reduce negative consequences, protect patients against adverse events, and promote patients’ ability to cope with this condition. Patients with high resilience can have a stronger capacity for self-reflection and a better tolerance of negative feelings in order to better cope with anxiety, depression, and psychological distress [55].

We found that social support reduced anxiety and depression among patients with post-COVID-19 syndrome. Also, our results show a significant positive relationship between social support and quality of life. A meta-analysis has already shown that post-COVID-19 syndrome reduces patients’ quality of life and maintains symptoms such as sleep disturbance, anxiety, depression, fatigue, and dyspnea [10]. Cohort studies suggest that the negative consequences of post-COVID-19 syndrome on patients’ quality of life and mental health remain even a year after infection [34,56]. Several studies including COVID-19 patients found that high social support had a negative association with anxiety and depression symptoms [57,58,59]. Moreover, social support positively affected COVID-19 patients’ quality of life during the COVID-19 pandemic [60,61]. Additionally, a systematic review confirmed the positive relationship between social support and people’s quality of life especially during the first wave of the pandemic [15].

Social support, or a social network, characterizes the functioning of individuals among other people, e.g., family members, friends, significant others, and neighbors [62]. During the pandemic, protective measures, such as quarantine, increased the loneliness of all citizens regardless of age group, with mental health consequences such as anxiety, depression, and suicide risk [29]. Following disasters, social support plays an essential protective role in maintaining mental health [63]. Similarly, several studies during the pandemic suggested that people could benefit from real-life and online social support [62,64,65,66]. Social support increased belongingness and community attachment during the pandemic and that resulted in reduced anxiety and depressive symptoms [67]. Additionally, peer and community support groups reduced psychological distress in the era of the pandemic [68]. Moreover, social support positively affected individuals’ self-efficacy during the pandemic, improving their ability to cope successfully with stressful experiences, such as compliance with quarantine and isolation measures [69,70,71]. Therefore, social support is an essential psychological resource that can improve mental health and quality of life in patients with post-COVID-19 syndrome, allowing them to successfully deal with the long-term psychological consequences of the disease.

Our study had several limitations. First, we used a convenience sample, which is not representative of the population of patients with post-COVID-19 syndrome in Greece. For example, most of our patients were females, while only a few had been hospitalized in a COVID-19 intensive care unit. Moreover, our sample was obtained from the long COVID Greece patient society. Thus, patients who did not belong to this society did not have the chance to participate in our study. Although we achieved the required sample size, further studies with random and bigger samples can add valuable information. Second, we used self-reported tools to measure resilience, social support, anxiety, depression, and quality of life in our patients. Thus, information bias is probable in our study. Especially for anxiety and depression, we need to use valid diagnostic criteria in order to obtain more robust results. In that case, longitudinal studies can be very helpful. Third, we assessed the independent effects of resilience and social support, eliminating some confounders, but other factors could also be possible confounders, e.g., socioeconomic status, educational level, family status, etc. Fourth, we performed a cross-sectional study measuring the variables at a specific time. Thus, a causal effect relationship between resilience and social support, and anxiety, depression, and quality of life cannot be established. Follow-up studies measuring changes over time could provide more valid results. Finally, we measured the impacts of two psychological factors (i.e., resilience and social support) on patients’ lives. Several other psychological variables (e.g., self-efficacy, mindfulness, optimism, loneliness, social integration, etc.) could affect patients’ mental health and quality of life. Thus, further studies should be performed in order to expand our knowledge in this field.

## 5. Conclusions

Our study identified a worrying proportion of patients with post-COVID-19 syndrome with anxiety and depression symptoms. Moreover, the quality of life among these patients was very poor. However, resilience and social support can be protective by reducing anxiety and depression and improving quality of life among patients with post-COVID-19 syndrome. The prevalence of psychological problems among patients with post-COVID-19 syndrome seems to be high. Since we are dealing with a new condition, our knowledge is very limited. Thus, the identification of factors that influence patients’ lives is crucial to reducing negative outcomes and improving quality of life.

Psychological resources, such as resilience and social support, are important for promoting positive adaptation in case of post-COVID-19 syndrome and reducing negative symptomatology. There is a need to provide on-time and updated psychological care services for patients with post-COVID-19 syndrome. Moreover, we should follow up on these patients for a longer period since post-COVID-19 syndrome can last for more than a year.

Patients with post-COVID-19 syndrome need time to adapt to their condition both physically and mentally. Since it is a new and unknown condition, patients should develop resilience over time in order to deal effectively with post-COVID-19 syndrome. Also, policymakers should develop and implement healthcare management programs to provide psychological support to patients.

Healthcare workers, especially clinical psychiatrists and psychologists, should be aware of the psychological needs of patients with post-COVID-19 syndrome in order to improve their mental health and quality of life. Moreover, healthcare professionals should carry out regular follow-up observations to assess the long-term effects of post-COVID-19 syndrome.

## Figures and Tables

**Table 1:** Demographic and clinical characteristics of patients with post-COVID-19 syndrome (N = 122).

Variable	N	%
Gender		
Females	89	73.0
Males	33	27.0
Age (years), mean, standard deviation	44.8	11.5
Hospitalization in COVID-19 ward		
No	101	82.8
Yes	21	17.2
Hospitalization in COVID-19 intensive care unit		
No	118	96.7
Yes	4	3.3
Duration of COVID-19 symptoms (months), mean, standard deviation	11.6	8.6
Anxiety disorders before post-COVID-19 syndrome		
No	99	81.1
Yes	23	18.9
Depression before post-COVID-19 syndrome		
No	108	88.5
Yes	14	11.5

**Table 2:** Descriptive statistics for the scales in our study.

Scale	Mean	Standard Deviation	Median	Minimum Value	Maximum Value
Patient Health Questionnaire					
Anxiety score	3.22	1.78	3	0	6
Depression score	3.56	1.69	4	0	6
EQ-5D-3L index value	0.36	0.36	0.31	−0.33	1
EQ-5D-3L VAS	54.10	21.71	55	0	100
Brief Resilience Scale	3.32	0.87	3.33	1.17	5
Multidimensional Scale of Perceived Social Support					
Family support	5.41	1.61	5.75	1	7
Friend support	5.22	1.56	5.63	1	7
Significant other support	5.73	1.38	6	1	7

**Table 3:** Univariate and multivariable linear regression analysis with anxiety score as the dependent variable.

Independent Variable	Univariate Model		Multivariable Model	
	Unadjusted Beta Coefficient (95% CI)	*p*-Value	Adjusted Beta Coefficient (95% CI) ^a^	*p*-Value
Gender (males vs. females)	−0.59 (−1.31 to 0.12)	0.10	−0.53 (−1.21 to 0.14)	0.12
Age (years)	−0.04 (−0.07 to −0.02)	0.003	−0.03 (−0.06 to −0.007)	0.01
Hospitalization in COVID-19 ward (yes vs. no)	0.94 (0.11 to 1.77)	0.03	0.96 (0.15 to 1.77)	0.02
Duration of COVID-19 symptoms	0.006 (−0.03 to 0.04)	0.77	−0.01 (−0.05 to 0.02)	0.46
Anxiety disorders before post-COVID-19 syndrome (yes vs. no)	1.17 (0.38 to 1.97)	0.004	0.55 (−0.33 to 1.43)	0.22
Depression before post-COVID-19 syndrome (yes vs. no)	0.80 (−0.20 to 1.80)	0.12	0.04 (−1.01 to 1.10)	0.94
Resilience	−0.87 (−1.20 to −0.53)	<0.001	−0.56 (−0.94 to −0.17)	0.005
Family support	−0.14 (−0.34 to 0.06)	0.17	0.22 (−0.03 to 0.47)	0.08
Friend support	−0.24 (−0.45 to −0.04)	0.02	0.04 (−0.20 to 0.28)	0.75
Significant other support	−0.40 (−0.62 to −0.17)	0.001	−0.34 (−0.64 to −0.03)	0.03

CI: confidence interval; ^a^ *p*-value for ANOVA < 0.001; R^2^ for the final multivariable model was 24.9%.

**Table 4:** Univariate and multivariable linear regression analysis with depression score as the dependent variable.

Independent Variable	Univariate Model		Multivariable Model	
	Unadjusted Beta Coefficient (95% CI)	*p*-Value	Adjusted Beta Coefficient (95% CI) ^a^	*p*-Value
Gender (males vs. females)	−0.81 (−1.48 to −0.13)	0.02	−0.78 (−1.44 to −0.12)	0.02
Age (years)	−0.03 (−0.06 to −0.004)	0.03	−0.02 (−0.04 to 0.008)	0.19
Hospitalization in COVID-19 ward (yes vs. no)	0.71 (−0.09 to 1.51)	0.08	0.66 (−0.14 to 1.45)	0.10
Duration of COVID-19 symptoms	0.01 (−0.02 to 0.05)	0.50	−0.005 (−0.04 to 0.03)	0.75
Anxiety disorders before post-COVID-19 syndrome (yes vs. no)	0.65 (−0.12 to 1.42)	0.09	0.14 (−0.73 to 1.00)	0.76
Depression before post-COVID-19 syndrome (yes vs. no)	0.34 (−0.62 to 1.29)	0.48	−0.25 (−1.29 to 0.79)	0.64
Resilience	−0.73 (−1.05 to −0.40)	<0.001	−0.42 (−0.80 to −0.05)	0.03
Family support	−0.21 (−0.39 to −0.02)	0.03	0.15 (−0.10 to 0.39)	0.23
Friend support	−0.29 (−0.48 to −0.10)	0.003	−0.02 (−0.26 to 0.22)	0.89
Significant other support	−0.45 (−0.66 to −0.24)	<0.001	−0.39 (−0.69 to −0.09)	0.01

CI: confidence interval; ^a^ *p*-value for ANOVA < 0.001; R^2^ for the final multivariable model was 19.9%.

**Table 5:** Univariate and multivariable linear regression analysis with EQ-5D-3L index value as the dependent variable.

Independent Variable	Univariate Model		Multivariable Model	
	Unadjusted Beta Coefficient (95% CI)	*p*-Value	Adjusted Beta Coefficient (95% CI) ^a^	*p*-Value
Gender (males vs. females)	0.19 (0.05 to 0.34)	0.01	0.12 (−0.02 to 0.26)	0.11
Age (years)	0.003 (−0.002 to 0.009)	0.24	0.0004 (−0.005 to 0.006)	0.88
Hospitalization in COVID-19 ward (yes vs. no)	−0.06 (−0.23 to 0.11)	0.50	0.008 (−0.16 to 0.18)	0.93
Duration of COVID-19 symptoms	−0.009 (−0.02 to −0.002)	0.01	−0.007 (−0.01 to 0.00003)	0.05
Anxiety disorders before post-COVID-19 syndrome (yes vs. no)	−0.25 (−0.42 to −0.09)	0.002	−0.08 (−0.26 to 0.11)	0.40
Depression before post-COVID-19 syndrome (yes vs. no)	−0.31 (−0.51 to −0.12)	0.002	−0.16 (−0.38 to 0.06)	0.15
Resilience	0.15 (0.07 to 0.22)	<0.001	0.10 (0.02 to 0.18)	0.01
Family support	0.03 (−0.01 to 0.07)	0.02	−0.04 (−0.09 to 0.01)	0.13
Friend support	0.03 (−0.01 to 0.07)	0.18	−0.03 (−0.08 to 0.02)	0.22
Significant other support	0.09 (0.04 to 0.13)	<0.001	0.10 (0.04 to 0.16)	0.002

CI: confidence interval; ^a^ *p*-value for ANOVA < 0.001; R^2^ for the final multivariable model was 23.7%.

**Table 6:** Univariate and multivariable linear regression analysis with EQ-5D-3L VAS as the dependent variable.

Independent Variable	Univariate Model		Multivariable Model	
	Unadjusted Beta Coefficient (95% CI)	*p*-Value	Adjusted Beta Coefficient (95% CI) ^a^	*p*-Value
Gender (males vs. females)	9.96 (1.35 to 18.57)	0.02	5.54 (−3.17 to 14.25)	0.21
Age (years)	0.15 (−0.19 to 0.49)	0.37	0.0001 (−0.34 to 0.34)	0.99
Hospitalization in COVID-19 ward (yes vs. no)	0.63 (−9.72 tο 10.98)	0.90	4.53 (−5.93 to 14.99)	0.39
Duration of COVID-19 symptoms	−0.45 (−0.89 to 0.0003)	0.05	−0.36 (−0.80 to 0.09)	0.11
Anxiety disorders before post-COVID-19 syndrome (yes vs. no)	14.27 (−23.92 to −4.61)	0.004	−6.52 (−17.92 to 4.89)	0.26
Depression before post-COVID-19 syndrome (yes vs. no)	−16.73 (−28.61 to −4.85)	0.006	−7.54 (−21.21 to 6.13)	0.28
Resilience	6.35 (1.99 to 10.71)	0.005	3.76 (−1.21 to 8.74)	0.14
Family support	1.79 (−0.62 to 4.21)	0.14	−1.55 (−4.80 to 1.70)	0.35
Friend support	1.38 (−1.11 to 3.88)	0.27	−1.82 (−4.97 to 1.34)	0.26
Significant other support	4.63 (1.93 to 7.34)	0.001	5.55 (1.59 to 9.51)	0.006

CI: confidence interval; ^a^ *p*-value for ANOVA < 0.001; R^2^ for the final multivariable model was 15.1%.

## Data Availability

The data presented in this study are available on request from the corresponding author.
